# The palaeobiology of high latitude birds from the early Eocene greenhouse of Ellesmere Island, Arctic Canada

**DOI:** 10.1038/srep20912

**Published:** 2016-02-12

**Authors:** Thomas A. Stidham, Jaelyn J. Eberle

**Affiliations:** 1 Key Laboratory of Vertebrate Evolution and Human Origins, Institute of Vertebrate Paleontology and Paleoanthropology, Chinese Academy of Sciences, Beijing 100044, China; 2 University of Colorado Museum of Natural History and Department of Geological Sciences, University of Colorado at Boulder, 265 UCB, Boulder, CO 80309, USA

## Abstract

Fossils attributable to the extinct waterfowl clade Presbyornithidae and the large flightless Gastornithidae from the early Eocene (~52–53 Ma) of Ellesmere Island, in northernmost Canada are the oldest Cenozoic avian fossils from the Arctic. Except for its slightly larger size, the Arctic presbyornithid humerus is not distinguishable from fossils of *Presbyornis pervetus* from the western United States, and the *Gastornis* phalanx is within the known size range of mid-latitude individuals. The occurrence of *Presbyornis* above the Arctic Circle in the Eocene could be the result of annual migration like that of its living duck and geese relatives, or it may have been a year-round resident similar to some Eocene mammals on Ellesmere and some extant species of sea ducks. *Gastornis*, along with some of the mammalian and reptilian members of the Eocene Arctic fauna, likely over-wintered in the Arctic. Despite the milder (above freezing) Eocene climate on Ellesmere Island, prolonged periods of darkness occurred during the winter. Presence of these extinct birds at both mid and high latitudes on the northern continents provides evidence that future increases in climatic warming (closer to Eocene levels) could lead to the establishment of new migratory or resident populations within the Arctic Circle.

Fossil birds from within the Arctic Circle are rare. The Cretaceous record is restricted to the flightless diving hesperornithiform birds from Devon Island^1^, Ellesmere Island^2^, and Alaska^3^, as well as bird tracks from Alaska^4^. The only known Paleogene record of Arctic birds is a report of *Gastornis* (*Diatryma* of some older workers; Gastornithidae) and potentially *Presbyornis* (Anseriformes) from the early Eocene (Wasatchian North American Land Mammal Age, NALMA) of Ellesmere Island, Canadian High Arctic^5^,^6^ (~79°N. lat.; see Fig. 1). In contrast, the late Cenozoic record is somewhat better^7^,^8^, but understudied in relation to the potential impacts of Pleistocene climate change on birds. As a step toward better understanding the history of birds in the Arctic, we describe the previously mentioned (but unpublished) *Presbyornis* and *Gastornis* material from Ellesmere Island^9^,^10^, and an additional avian pedal phalanx that has not been discussed before.

Beginning in the 1970s, paleontologists Mary Dawson, Robert (Mac) West, Malcolm McKenna, and J. Howard Hutchison collected fossil vertebrates from the Paleogene sequence of the Eureka Sound Group exposed on Ellesmere Island, and more recent expeditions have added to the accumulated diverse vertebrate fauna, including fish, turtles, crocodilians, many clades of mammals ranging from primates to brontotheres, and birds^5^. In an early report on the fossil vertebrates recovered from Ellesmere Island, the authors mentioned the presence of *Gastornis* (*Diatryma*) and a *Presbyornis*-like bird^9^. Those specimens remain the only avian material recovered from the Eocene sequence on Ellesmere, and are the only avian fossils from the Paleogene of the High Arctic (representing the northernmost Paleogene avian records in North America). The *Gastornis* material reported from Ellesmere Island is the northernmost record of that taxon in North America^10^. However, the Paleogene avian material from Ellesmere has not been described or illustrated. The osteological terminology follows Baumel and Witmer^11^ with English equivalents of the Latin terms.

Eocene terrestrial vertebrates are known from multiple sites on Ellesmere Island, but the best documented and most diverse faunal assemblage, including the bird fossils described here, is from strata of the Eureka Sound Group exposed near Bay Fiord on central Ellesmere Island (Fig. 1). Although Paleogene fossil-bearing rocks were first recognized in the Canadian Arctic over a century ago^12^, the Eureka Sound Group was not named until 1950 by Troelsen^13^. Subsequently, the Eureka Sound was reduced to a formation by Tozer^14^, and remained assigned to that rank until it was raised formally back to a group by Miall^15^, where it has since remained^5^. Three sets of names have been applied to strata of the Eureka Sound Group. Following their discovery of Paleogene vertebrates on central Ellesmere Island^15^,^16^, West *et al.*^17^ subdivided the Eureka Sound Formation into four informal members with the uppermost Member IV containing the Eocene terrestrial vertebrates. Independently, Miall^15^ and Ricketts^18^ raised the Eureka Sound Formation to group rank and subdivided it into formations. Consequently (and confusingly), Paleogene terrestrial vertebrate-bearing strata on central Ellesmere Island fall under three different names – Member IV of West *et al.*^19^, the Margaret Formation of Miall^15^, and the Iceberg Bay Formation of Ricketts^18^.

Today’s general consensus is that the Eureka Sound is a group divisible into numerous formations, and generally Miall’s^15^ formational names are applied to Ellesmere Island, while Ricketts’s^18^ nomenclature is most often used on nearby Axel Heiberg Island^5^. Following this usage, we assign the Eocene fossil bird localities (and related stratigraphic horizons) discussed below to the Margaret Formation.

The Margaret Formation comprises coarsening-upward sequences of interbedded cross-bedded sandstone, siltstone, mudstone, and coal that are interpreted as proximal delta-front to delta-plain paleoenvironments characterized by abundant shoreline sands, alluvial to estuarine channels, coal swamps, lagoons and bays, and well-forested, low-gradient interfluves^15^,^18^,^20^,^21^.

At Bay Fiord, Eocene vertebrate fossils occur in two fossil assemblages in the Margaret Formation that are separated by a 478 m thick stratigraphic gap in the fossil record^21^. The stratigraphically lower of the two preserves a diverse early Eocene (late Wasatchian NALMA; ca. 52–53 Ma) fauna that contains fishes, amphibians, alligators, a snake, nine families of turtle, at least 25 genera of mammals^5^, and the bird fossils described below. The late Wasatchian NALMA fauna is likely within the Early Eocene Climatic Optimum (EECO), which marked the height of global warmth since the extinction of the dinosaurs^22^. The second, stratigraphically higher fossil assemblage is considerably less diverse (lacking avian material at present), with a handful of Bridgerian NALMA^23^ (ca. 47–51 Ma) taxa, including the early brontothere *Palaeosyops*^21^.

The gastornithid, presbyornithid and indeterminate avian phalanx specimens described below were collected by J. Howard Hutchison from localities ELS76-85, ELS76-42 and ELS76-43, respectively. Fieldwork at Bay Fiord in 2010 concluded that ELS76-42 and ELS76-43 are on the same stratigraphic horizon within the lower fossil assemblage at approximately the 1420–1425 m level on the composite stratigraphic section of Eberle and Eberth^21^, and ELS76-85 is the most productive Eocene vertebrate locality in the Arctic (stratigraphically higher than the other localities at 1437.5 m)^21^. The stratigraphically lower avian localities are about 805 meters above the contact between the Margaret and underlying Mt. Moore Formation (Eberth, pers. comm. to JE, 2015).

## Results

### Systematic Palaeontology

Family Gastornithidae Fürbringer 1888

*Gastornis* sp.

Material. Canadian Museum of Nature (Ottawa, Canada) CMN 32412. The specimen is a nearly complete pedal phalanx from locality ELS76-85, Margaret Formation, Eureka Sound Group, central Ellesmere Island, Nunavut, Canada (early Eocene; late Wasatchian NALMA).

Description. CMN 32412 lacks the stouter and more trapezoidal outline of more distal phalanges of *Gastornis*^24^,^25^, and has the broadened articular end present in the most proximal phalanx. The proximal dorsal to dorsolateral portion of the specimen is missing, and there is no evidence of a dorsoplantarly-oriented ridge on the articular surface that would have fit into the trochlear furrow. The proximal end is wider (28 mm, broken) than the distal end (24.8 mm). The width of the proximal end would have been greater than 44% of the length of the bone (62.2 mm; since it is damaged). The lateral edge of the bone is relatively straight (dorsal view); while the medial edge is slightly more concave. The plantar lip of the lateral collateral ligament fossa (the lateral plantar rim of the trochlea) is visible in dorsal view (extending lateral to the shaft) and continues proximally about one-third the length of the bone. In contrast, the equivalent medial part of the trochlea is not visible in dorsal view. The trochlea has a shallow trochlear furrow, and the lateral trochlear ridge extends slightly proximal to the medial ridge on the plantar surface. The lateral collateral ligament pit is large and deepest distoplantarly, and the medial pit is nearly circular in outline. In addition in lateral view, the plantar surface of the bone is concave, with the trochlea and condylar articulations being dorsoplantarly wider than the shaft.

Order Anseriformes Wagler 1831

Family Presbyornithidae Wetmore 1926

*Presbyornis* cf. *P. pervetus* Wetmore 1926

**Figure 2:**
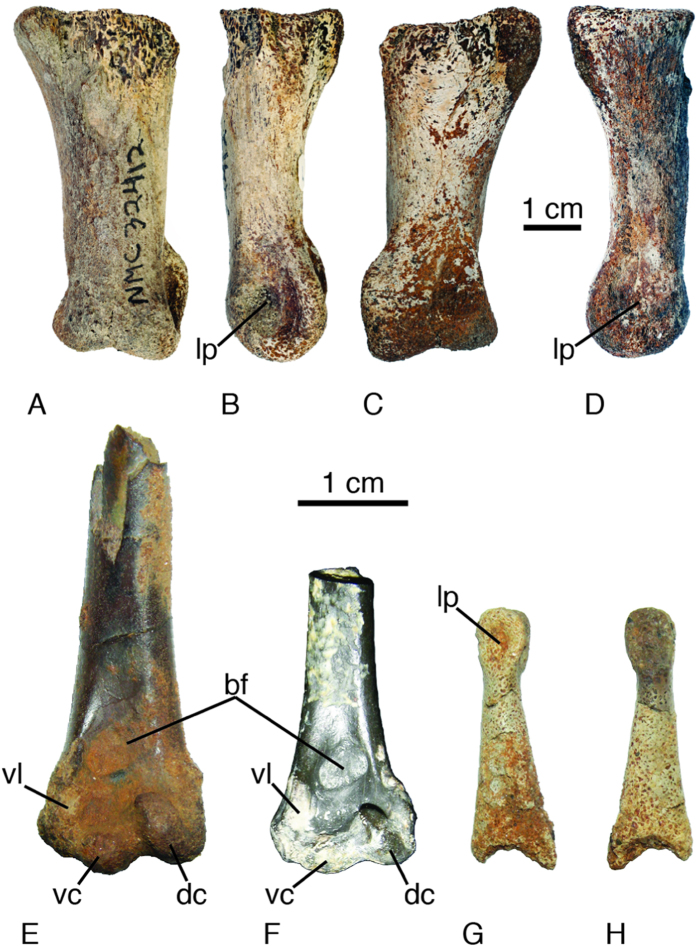
The Ellesmere Island avian fossils. The (probable) left proximal phalanx of digit IV from *Gastornis* sp. (CMNFV 32412): (**A**) dorsal view, (**B**) lateral view, (**C**) plantar view, and (**D**) medial view. The distal humeri of *Presbyornis*: (**E**) CMNFV 53369 from the Margaret Formation of Ellesmere Island, Canada, and (**F**) University of California Museum of Paleontology UCMP 119395 from the Wasatch Formation of Wyoming, USA in cranial view. The (**G**) right and (**H**) left surfaces of the indeterminate avian pedal phalanx (CMNFV 53368) showing the extensive sand pitting on the surface. Abbreviations: bf, brachial fossa; dc, dorsal condyle; lp, collateral ligament pit; vc, ventral condyle; and vl, facet for the ventral collateral ligament on the ventral supracondylar tubercle.

Material. CMNFV 53369. The specimen is the distal approximately one-third of a left humerus (Fig. 2), from locality ELS76-43 (=77H7-9-3), Margaret Formation, Eureka Sound Group, central Ellesmere Island, Nunavut, Canada (early Eocene; late Wasatchian NALMA).

Description. The bone is dark brown in color, and it exhibits some sand-induced surface pitting. That pitting is more pronounced on the distal end of the bone. Despite that overprint, the surface of the bone appears to have been smooth, indicating that the specimen likely is from an osteologically adult individual^26^. The proximal end of the shaft is subcircular in cross-section, but slightly wider medio-laterally.

From the proximal end towards the distal end of the cranial surface, the shaft flattens from a distinctly convex surface proximally. There is a distinct brachial (muscle) fossa that is deepest ventrodistally and shallows dorsally. The fossa’s distal end has a distinct border. Overall, the fossa is ovoid in outline with its long axis oriented dorsoproximal to ventrodistal. The distal edge of the brachial fossa is at the same proximodistal level as the proximal end of the ventral collateral ligament attachment at the apex of the ventral supracondylar tubercle. Just ventral to the proximal end of the apex of the supracondylar tubercle is a poorly preserved muscle scar pit (attachment of pronator brevis of Howard^27^ that is equivalent to the origination of the *m. flexor carpi ulnaris* of Livezey^28^) that is separate from the ventral collateral ligament attachment. The ventral collateral ligament scar on the supracondylar tubercle is proximodistally elongate, and its dorsal margin is relatively straight. The ventral condyle extends distal to the dorsal condyle. The area distal to the brachial fossa and proximal to the condyles is flat to slightly concave. The dorsal condyle extends proximally to the same level as the proximal end of the ventral collateral ligament scar. The long axis of the outline of the dorsal condyle is not parallel to the long axis of the humerus because the distal end is located dorsal to the proximal end.

The shaft is convex in caudal view. The olecranon fossa is relatively small and restricted to the area immediately caudal to the ventral condyle. The fossa is deepest ventrally. The distal end of the bone is worn, and there is a slight hint of a shallow scapulotriceps groove at the most distal part of the caudal side. The dorsal epicondyle has a pair of muscular pits (present in other anseriforms), but they are poorly preserved because the division between the two pits cannot be discerned. The dorsal epicondyle extends further proximal than the supracondylar tubercle/collateral ligament scar. The ventral side of the specimen is poorly preserved and no muscle scars are visible.

Aves indet.

Material. CMNFV 53368, a phalanx (Fig. 2) from locality ELS76-42 (=76H7-10-10), Margaret Formation, Eureka Sound Group, central Ellesmere Island, Nunavut (Early Eocene; Wasatchian NALMA).

Description. The specimen appears to be the proximal phalanx likely from digit III of the left side. The bone is off-white to gray in color and is deeply pitted all over its surface. The pitting is caused by sand grains that were embedded in the bone surface, and does not indicate an early ontogenetic age (Fig. 2). The proximal end is mediolaterally crushed/flattened, and the left side (dorsal view) of the distal end is broken off. The proximal articulation maintains a central ridge for articulation with the tarsometatarsus. In lateral view, the articulation is C-shaped. The distal end (lateral sides) has a deep collateral ligamental pit. The furrow for the articulation with the next more distal phalanx extends dorsally and plantarly to about the same proximal level and that level is proximal to the ligamental pits. The plantar side appears to have been flatter than the dorsal (convex) side, but the crushing of the specimen has distorted the shape.

Comparisons.

The length of the Ellesmere *Gastornis* specimen (62.2 mm) is less than the largest specimens of *Gastornis* from North America (68.6–80.1 mm) and that from Louvois, France (75.9 mm)^25^. The morphology of the Ellesmere specimen is similar to that reported and illustrated for the proximal phalanx of digit IV of *Gastornis* from Louvois^25^, Wyoming^24^, Monthelon^29^, and New Jersey^30^, but the plantar ridges mentioned in some *Gastornis* specimens appear to be absent in this specimen. The proximal widening of the articular region (in dorsal view) is similar across specimens. The Ellesmere specimen lacks the medially expanded plantar rim of the trochlea (plantar lip of the collateral ligament pit) that is visible in dorsal view in the Paleocene Louvois specimen^25^, and that state appears absent in other *Gastornis* specimens^24^,^29^. Furthermore, the plantar lip of the collateral ligament pit in CMN 32412 extends relatively more proximal than the state in the Louvois specimen^25^, but is similar to *Gastornis* from Wyoming^24^. The length of the phalanx and the ratio of the proximal width to the length of the bone (above) are within the ranges reported for *Gastornis*^25^.

The variation in the morphology of the humerus seems to be within that seen among the abundant Wyoming specimens of *Presbyornis pervetus* (Fig. 2). There are no apomorphies (versus other Paleogene anseriform taxa) in the distal humerus for *Presbyornis*, but there also are no character differences between the Ellesmere Island fossil and specimens of *Presbyornis pervetus* from Wyoming. The Ellesmere specimen was directly compared to casts of *Presbyornis pervetus* from Wyoming. The postcranial skeleton of *Presbyornis* is notoriously plesiomorphic^31^,^32^. The same shape, depth, and overall morphology of the brachial fossa can be seen among *Presbyornis* specimens from more southerly locations (Fig. 2). However, the Ellesmere specimen is on the larger end of the size range present in Wyoming (Table 1), and the Ellesmere Island specimen also is within the known stratigraphic range of *Presbyornis* in Wyoming^5^,^33^ (i.e., Wasatchian NALMA). There are no characters that appear to separate the Ellesmere humerus from what is currently recognized as *Presbyornis pervetus*, but also no clear derived characters to unite them into a clade.

Various authors have suggested that the morphological variation exhibited among the Wyoming *Presbyornis* bone assemblages might represent multiple closely related species^33^,^34^, but at present no one has discerned a way to separate any potential species within the *Presbyornis pervetus* collections from North America. In contrast, an early Eocene species of *Presbyornis*, *P*. *mongoliensis*, has been described from Mongolia^35^, and it likely does represent a species different from the penecontemporaneous North American *Presbyornis pervetus*. That Mongolian material also is smaller than the Ellesmere specimen^35^ (Table 1). Thus, it is possible that the Ellesmere specimen represents a taxon separate from *Presbyornis pervetus*, but there is no way at present to determine that status. Furthermore, the slightly larger size of the Ellesmere specimen compared to individuals at lower latitudes could be the result of Bergman’s Rule, and that cline has been proposed for the Arctic and mid-latitude mammal *Heptodon* from the Eocene^21^.

The indeterminate avian phalanx is consistent with *Presbyornis* (clearly lacking any autapomorphies), but it also easily could belong to another taxon of bird (given its somewhat more robust morphology), though its size and morphology is certainly inconsistent with that of the much larger *Gastornis*, which has stouter and more robust pedal phalanges (Fig. 2). In contrast to the *Presbyornis* specimen, the *Gastornis* phalanx is not larger than individuals known from further south in North America, but is within the known size variation for the group. The variation in size among *Gastornis* individuals has been attributed to both species differences and the possibility of sexual dimorphism^25^.

## Discussion

The early Eocene vertebrate fauna from Ellesmere Island is from a unique temporal interval of mild mean annual temperatures and above-freezing winters that allowed crocodilians and turtles to survive above the Arctic Circle. Today, the Eocene vertebrate localities on central Ellesmere are at nearly 79°N latitude, and during the Eocene, they were just a few degrees further south^36^,^37^. The fossils and sedimentology indicate a lush, rain forest community on a coastal delta plain. Multiple palaeoclimate proxies, ranging from oxygen isotope analysis of vertebrate bones and teeth to palaeofloral analyses, estimate a mild temperate climate for the Eocene High Arctic, where winters remained at or just above freezing and summer temperatures extended to 20 °C or higher^5^. These temperatures are a far cry from today’s High Arctic, where central Ellesmere Island experiences a mean annual temperature of −19 °C, a warm month mean temperature of about 6 °C and a cold month mean temperature of −38 °C or colder^38^.

Despite the mild Eocene Arctic climate, the vertebrate fauna would have experienced months of total darkness and cooler temperatures during the winter. Recent isotopic work suggests that some mammals, including the hippo-like *Coryphodon*, were year-round residents in the High Arctic^39^. Given that *Gastornis* was large (approaching 2 m) and flightless, it likely also was a year-round resident of the Arctic. In contrast, the volant *Presbyornis* might have been a seasonal migrant to the Arctic. The previously reported geographic distribution of *Presbyornis pervetus* was restricted to Utah, Wyoming, and Colorado^33^ with all records (USA and Mongolia) between 37° and 45°N. latitude, as compared to the 79°N. latitude of the new specimen that is greater than 4000 km further north (with a similar geographic disparity for specimens of *Gastornis* in the early Eocene of North America).

Extant galloanserines include migratory species, and migration in birds is (at least in part) genetically controlled^40^,^41^. Therefore, *Presbyornis*, which is phylogenetically within the crown group of galloanserines^31^,^32^,^34^, should have had the genetic predisposition for migration and the ability to migrate, given its skeletal morphology. However, it is unknown whether those genes were expressed in any presbyornithid because both migratory and non-migratory species (and populations) are present among various species of extant galloanserines^42^. The best data that would help support (though not prove) a migratory lifestyle for *Presbyornis* would be the discovery of juvenile specimens in the Arctic. However, both of the Ellesmere specimens reported above are from osteologically adult individuals. Furthermore, migratory behavior is not a requirement for a large geographic range because many extant non-migratory species have large geographic ranges (e.g., Osprey, Barn Owl, and Ostrich). However, as a filter-feeding bird, *Presbyornis* likely would have had a difficult time obtaining food during the winter decrease in (primary) productivity that occurred during the extended dark and low light periods of the Paleogene Arctic winters.

In contrast to migration, some galloanserine birds today (such as Ruffed Grouse, Spruce Grouse, Sharp-tailed Grouse, Willow Ptarmigan, and Rock Ptarmigan) are Arctic residents^43^, and even some waterfowl winter above the Arctic Circle^44^. Those waterfowl are able to forage and survive winters today that are much harsher than those reconstructed for the early Eocene of Ellesmere (see above). Ducks (Anatidae) of many lineages forage at night^44^,^45^, and extinct waterfowl species presumably would have been able to forage during the dark winters of the Eocene Arctic. Recent work^46^ has demonstrated greater tetrapod foraging activity and greater biological activity during the Arctic winter than previously known, hinting that such levels of activity should extend into the deep past. Even though individual waterfowl survive wintering above the Arctic Circle, obtaining food is difficult, and a variety of hypotheses have been erected as to how the individual birds compensate for the loss of daylight and reduction in prey availability^44^. Despite those challenges, it is possible that the occurrence of *Presbyornis* in the Arctic Eocene may have been as a resident, as closely related galloanserine birds are able to survive the much harsher Arctic winters today.

The current geographic ranges of migratory birds are shifting towards higher latitudes, and climatic warming has been implicated in phenological changes among birds^47^,^48^. Migrating and breeding at higher latitudes have been correlated with a reduced predation risk^49^. It is possible that the far northern occurrence of *Presbyornis* in the Arctic Circle could be either the result of a migratory population or alternatively a northern geographic expansion paralleling the pattern observed for many extant birds over the last century of warming. If anything, species of *Presbyornis* likely were affected by warming events in the early Eocene parallel to the response of extant birds to current warming. In contrast, the large flightless *Gastornis* definitely was an Arctic resident. The diet of gastornithids is debated and varies between carnivory and some type of durophagous herbivory^50^,^51^, but the most recent (isotopic and morphological) data support herbivory. Palynological and macrofloral studies indicate lush, early Eocene Arctic forests, comparable in richness to today’s swamp-cypress and broadleaf floodplain forests in the southeastern United States^5^,^52^, and like the herbivorous mammals on Ellesmere Island in the Eocene, *Gastornis* would have had the same access to dietary materials through the winter that kept the resident mammals alive. That the jaw anatomy (and inferred adductor musculature) of *Gastornis* suggests that it fed on tough plant material^51^ fits well with the Arctic winter forage that was available to the large mammalian herbivores, including wood and leaf litter, fungi, and evergreen conifers^39^.

Gastornithidae is Holarctic in distribution and *Presbyornis* (*P*. *pervetus* and *P*. *mongoliensis*) is known from North America and Asia during the Early Eocene^33^,^35^,^53^. Purported records of presbyornithids from Europe are not well supported^54^. The geographic and stratigraphic distribution of those two bird clades suggests that intercontinental dispersal (rather than intercontinental vicariance) likely was the process resulting in their widespread geographic distribution in the Northern Hemisphere^55^. Dispersal across the northern continents via high latitude land corridors has been widely hypothesized for mammalian clades with similar geographic and temporal distributions, and those dispersals have been tied to warming climates and the opening of northern high latitude land bridges^56^,^57^. The Holarctic distribution of Gastornithidae is similar to several early Eocene mammalian genera, including *Coryphodon*, the rodent *Paramys*, the mesonychid *Pachyaena*, and the carnivoran *Miacis*, which inhabited mid- and high latitude North America, as well as Asia and Europe in the early Eocene, and the North American/Asian distribution of *Presbyornis* parallels that of several Arctic genera, including the hyaenodontid creodont *Prolimnocyon*, the brontotheres *Eotitanops* and *Palaeosyops*, and the tapiroids *Heptodon* and *Homogalax* that occur only in North America and Asia (not Europe)^58^,^59^,^60^. Those biogeographic similarities between birds and mammals firmly places birds within the hypotheses of climate-related intercontinental dispersals in the early Eocene.

Occurrence of a diversity of terrestrial and aquatic vertebrate taxa in early Eocene-aged strata on Ellesmere Island indicates that a complex Arctic ecosystem was present during that time, and that productivity must have been high enough to sustain those higher trophic levels of vertebrates, even during the darkness that occurred through part of the Eocene winter. Given the geological and paleontological background (on Ellesmere Island and elsewhere), it is plausible that the current trend of climatic warming in the 20th and 21st centuries could lead to the establishment of Arctic resident populations of tetrapod species that currently occur much further south, as well as facilitate the intercontinental dispersal of avian clades among the continents of the Northern Hemisphere.

## Additional Information

**How to cite this article**: Stidham, T. A. and Eberle, J. J. The palaeobiology of high latitude birds from the early Eocene greenhouse of Ellesmere Island, Arctic Canada. *Sci. Rep.* **6**, 20912; doi: 10.1038/srep20912 (2016).

## Figures and Tables

**Figure 1:**
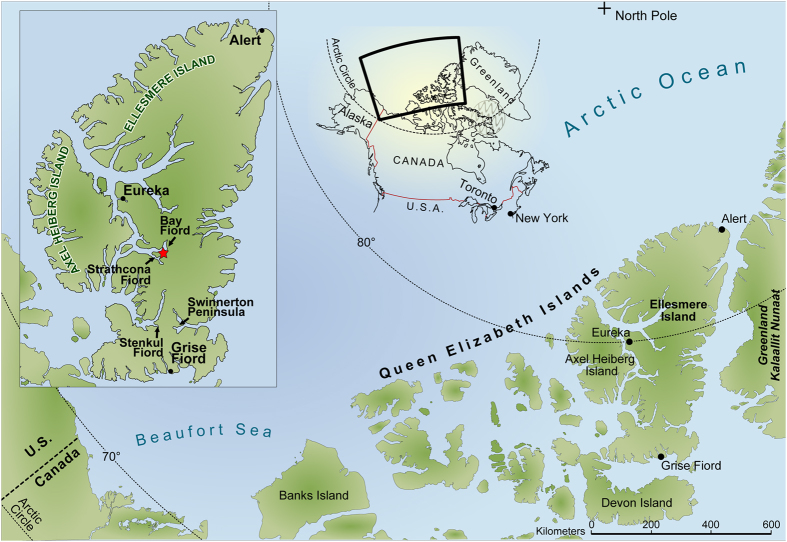
Map of northern Canada north of the Arctic Circle showing principal locations where Eocene terrestrial vertebrates have been recovered on Ellesmere Island (inset)–Stenkul Fiord, Swinnerton Peninsula, Strathcona and Bay Fiords. Star indicates the location of the Bay Fiord fossil-collecting area which preserves the most diverse Eocene vertebrate assemblage from the Arctic, including the bird fossils described in the text. Figure modified from Eberle and Greenwood^5^.

**Table 1:** Measurements (in mm) of the distal end of the humerus of *Presbyornis* specimens.

Specimen	Dorsoventral Width	Craniocaudal Depth
CMNFV 53369	15.1	8.8
UCMP 119394	14.4	7.6
UCMP 119395	14.0	7.4
UCMP 119396	13.6	7.2
UCMP 119397	14.0	7.0
UCMP 119398	11.4	6.1
UCMP 119399	13.0	7.3
UCMP 119400	13.5	6.7
UCMP 119401	12.5	6.6
Mongolian Specimens	9.7–13.2	–

UCMP is the University of California Museum of Paleontology (Berkeley, USA). The range of distal width measurements for the Mongolian material is from Kurochkin and Dyke^31^.
